# Ambivalent and heavy burdened wanderers on a road less travelled: a meta-ethnography on end-of-life care experiences among family caregivers in rural areas

**DOI:** 10.1186/s12913-024-11875-3

**Published:** 2024-12-21

**Authors:** Elisabet Breivik, Bente Ervik, Gabriele Kitzmüller

**Affiliations:** 1 Department of Health and Care Sciences, Faculty of Health Sciences, UiT, The Arctic University of Norway, Tromsø, Norway; 2 Department of Oncology, University Hospital of North Norway, Tromsø, Norway

**Keywords:** Meta-ethnography, Experiences, Rural, Family, Cancer, Palliative care, End of life

## Abstract

**Background:**

As the population ages, more people will be diagnosed with cancer, and they will live longer due to receiving better treatment and optimized palliative care. Family members will be expected to take on more responsibilities related to providing palliative care at home. Several countries have expressed their vision of making home death an option, but such a vision can be more challenging in rural areas. There is a lack of synthesized research providing an in-depth understanding of rural family caregiving for people with cancer at the end of life. Thus, the purpose of this study was to synthesize and reinterpret the findings from qualitative research on rural family caregivers of adult cancer patients at the end of life.

**Methods:**

We conducted a meta-ethnography following Noblit and Hare’s approach. A systematic literature search of four databases and extensive manual searches were completed in April 2022. The final sample included twelve studies from six different countries published in 2011–2022.

**Results:**

Based on the translation and synthesis of the included studies, four themes were developed (1) providing family care at the end of life in rural areas—a challenging endeavour; (2) the heavy responsibility of rural caregiving—a lonesome experience; (3) working on and behind the scenes; and (4) the strong and weak spots of community connectedness in rural areas. An overarching metaphor, namely, “ambivalent and heavy burdened wanderers on a road less travelled”, provides a deeper understanding of the meaning of rural family caregiving at the end of life.

**Conclusions:**

This study provides valuable insights into end-of-life cancer care for rural families on four continents. It is crucial to prepare family caregivers for the demanding role of palliative caregiving in rural areas. To address the long distances and poor access related to specialized health care services, outpatient palliative teams tailored to the families’ individual needs should be provided. In addition, more telehealth services, palliative units, or beds in local nursing facilities may reduce the number of exhausting trips that need to be made by caregivers and patients. Healthcare workers in rural areas need further education in palliative care.

**Trial registration:**

The study was registered in PROSPERO.

## Background

Approximately 56,8 million people worldwide need palliative care, of which 34% have cancer, but only 14% get such services [1]. Several definitions of palliative care exist. According to Radbruch et al., [2] palliative care is defined as the active, holistic care of individuals across all ages with serious health-related suffering due to severe illness, especially those near the end of life. It aims to improve the quality of life of patients, their families and their caregivers ([2], p.755). The European Association for Palliative Care (EAPC) emphasises that palliative care is interdisciplinary, includes the care of the patient and their family, and should be available in hospitals, hospices, and the community [3]. In our study, palliative care focuses on care provided for persons with incurable cancer during the last months or weeks.

About half of the world’s population have their homes in rural and remote areas [4]. The definition of “rural” may differ depending on factors such as geography, population density, and proximity to healthcare services [5].

Over the past few decades, families of persons with cancer have increasingly played a vital role in providing home-based palliative care [6, 7]. As treatment has improved, people are living longer with cancer, and with the ageing population, the demand for palliative care is increasing due to more complex end-of-life situations [8] and due to frailty and comorbidity in the elderly [4]. While early palliative care is recommended [9], rural areas often lack access to services [9, 10], and healthcare professionals’ knowledge on palliative care is often limited [8].

Although the World Health Organization (WHO) [1] has declared palliative care to be a human right, access to palliative care is unevenly dispersed and often concentrated in urban areas [4]. Studies from various regions worldwide indicate that most individuals prefer receiving palliative care and dying in their homes [11–13]. However, there is a discrepancy between the preferred and actual place of death [14]. Living in rural areas often requires relocating to nursing home facilities or hospitals to access end-of-life palliative care [15]. End-of-life decisions regarding the preferred place of death depend on several factors; for family caregivers, it is important to feel safe and supported when fulfilling the care recipient’s wish to die at home [12].

There are significant barriers to accessing palliative care services due to shortages of health professionals and services, limited access to specialists, and a lack of interprofessional teamwork [16, 17]. Additionally, disparities in access to palliative care services exist between urban and rural areas [15, 18], and family caregivers in rural areas face more unmet support needs than those in urban areas [19]. The quality of palliative care for cancer care recipients also differs in rural and urban areas, underlining the necessity for further research in rural areas [16, 20].

Family caregivers, also known as informal caregivers, are unpaid individuals such as a spouse, partner, family member, friend, or neighbour, who help with activities of daily living and medical tasks [21]. Providing palliative care at home can be a major challenge for family caregivers living in rural areas. These individuals often face long distances to hospitals and health services, limited public transport, and inadequate road infrastructure [14]. In addition, they are struggling with managing personal hygiene and nutrition, administering pain management, and coordinating healthcare services [9, 14]. These challenges are considered burdensome [7] and reduce their quality of life [22]. Family caregivers are at risk of developing depression, anxiety, fatigue, and insomnia [23]. They experience more stress and anxiety than care recipients [23]. Especially towards the end of the care recipient’s life, caregivers experience health and emotional problems [24], and their mortality risk is also higher compared with other populations [25].

Family caregivers require information on the availability and accessibility of palliative services, and they also need practical and emotional support [26]. This is particularly important for those involved in end-of-life care during the final stages of life [27]. According to a recent survey, providing sufficient information alone is not enough to support family caregivers. Instead, a more empowering approach is needed to boost caregivers’ self-efficacy [24]. However, interventions that have been developed to support caregivers in rural areas have not yet had a significant impact on their well-being [10].

Although there have been reviews and meta-ethnographies that have synthesized the extant knowledge on home-based palliation and end-of-life care, rural areas have not been their main focus [11, 28]. Additionally, different perspectives have been blended, which has concealed the perceptions of family caregivers [17, 29]. The heterogeneity across studies makes it difficult to draw any firm conclusions about effective support strategies for these caregivers [10]. However, culturally appropriate delivery methods and palliative care education seem to be essential strategies for supporting caregivers in rural areas [4].

More research is needed to increase our understanding of the challenges and needs faced by families in rural areas that are supporting someone at the end of life [5, 10, 17] to better organize rural palliative end-of-life care [11]. In this study, we address the lack of meta-ethnographies on rural palliative caregiving for individuals with cancer at the end of life to arrive at possible directions for future practise, research, and health policies. Therefore, the purpose of the work described here was to synthesize and reinterpret findings from qualitative studies focusing on the experiences of rural family caregivers of adult persons with cancer at the end of life.

## Methods

In this study, we applied Noblit and Hare’s [30] meta-ethnographic approach to synthesize and reinterpret qualitative research on the topic. Meta-ethnography is a qualitative evidence synthesis methodology that is frequently used by healthcare professionals [31], for which reporting guidelines do exist [32]. The meta-ethnographic approach seeks to produce an integrated analysis of qualitative research on a particular topic, treating each paper as if it were a transcribed interview from a piece of primary research [30, 32]

Noblit and Hare’s 7-phase process for conducting meta-ethnography involves identifying key concepts from different studies and translating them into each other to demonstrate similarities (reciprocal translation) and differences (refutational translations). This process helps one arrive at a higher-level interpretation called a line of argument synthesis [30]. Translating studies into each other is a unique component of creating a synthesis, which is distinguished from other meta-synthesis approaches. To report our study, we followed the eMERGe meta-ethnography reporting guidance [32] based on Noblit and Hare’s approach [30]. The eMERGe reporting guidance is designed to enhance the quality of meta-ethnography reporting and improve the validity of the research process [32].

A study protocol was registered in PROSPERO 30.05.2022 (registration number: CRD42022332489) to avoid the duplication of work, improve the transparency of review procedures, minimize selective reporting, and increase quality [33]. To ensure high-quality research and relevant reviews, it is important to involve user representatives who have relevant experience [34]. In line with this knowledge, we invited a family caregiver who has experience with end-of-life palliative cancer care in a rural area to be involved in the current study. Her involvement provided a valuable first-person perspective on the interpretation of the findings.

### Search strategy: screening and outcomes

The meta-ethnography adhered to the Preferred Reporting Items for Systematic Reviews and Meta-Analyses (PRISMA) guidelines [35], and the search strategy was informed by the research aim, the research question, the research objectives and the meta‐ethnography purpose [36]. We applied a selective sampling strategy to identify all relevant studies within specified limits. A pilot search was conducted in relevant databases with the keywords *palliative care* combined with *families, rural,* and *qualitative methods.* A derivative formulation of Population, phenomenon of Interest and Context (PICo) for qualitative questions has been used [37] to clarify the research focus and the inclusion and exclusion criteria.

**Fig. 1:**
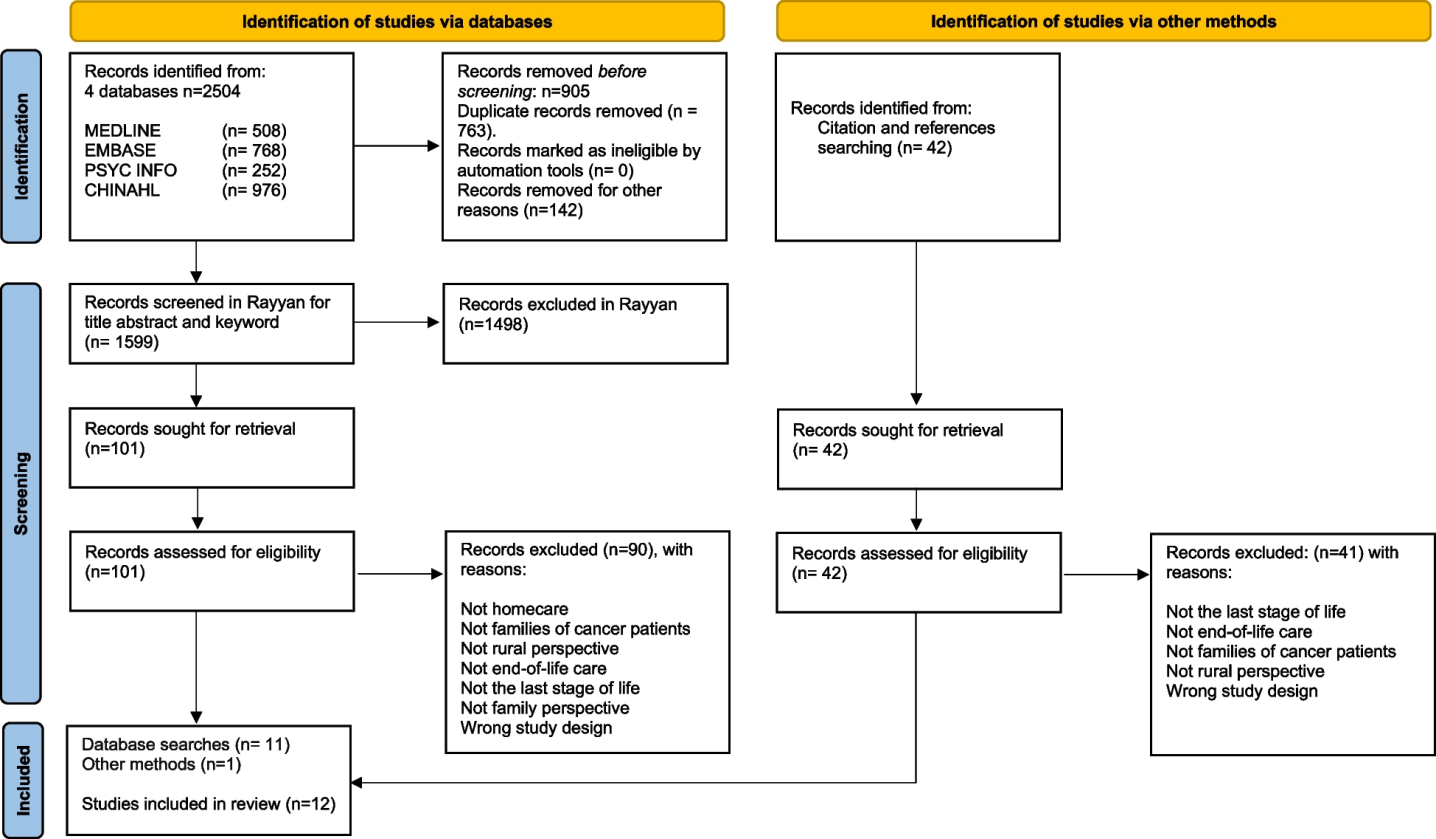
PRISMA flow diagram of study selection: indicating the number of studies identified by the search strategy, the number of studies excluded and included during screenings, and the final number of studies included. From: [35]

In cooperation with an expert librarian, relevant databases and keywords were identified, and a search strategy was developed. Systematic literature searches were conducted from February to April 2022 utilizing the following four electronic databases: PsycINFO, EMBASE (Ovid), MEDLINE (Ovid), and CINAHL. Our search string was created using a combination of keywords and Boolean operators (OR/AND) to ensure thoroughness. We continuously updated the searches throughout 2023. The chosen databases provide research articles in the field of health sciences [31]. Combinations and variations in subject terms, thesaurus and free text searching were modified to fit the different databases. Search limits were set to peer-reviewed journals written in English and Nordic languages published between January 2011 and December 2022. The search identified 2504 records that were downloaded into EndNoteX9, after which duplicates and records with incorrect publication forms were removed. A total of 1599 papers remained after this process (Fig. 1).

Records were further imported into the Rayyan Systematic Literature Review web tool [38] and screened against the eligibility criteria. Based on their title and abstract, 1498 articles were excluded. The first author performed the screening process in Rayyan in close dialogue with the third author. Following this initial screening process, both authors read all identified articles in a full-text reading to determine if the studies met the inclusion criteria. Any disagreement was discussed with all authors until a consensus was reached. One hundred and one articles were identified for full-text reading, and 90 were excluded for reasons in line with the eligibility criteria (Fig. 1). Ultimately, eleven articles from the database searches were included in the current study.

Additional search strategies were applied, such as hand searching in journals, screening the reference lists of already included studies, using citation checks in Google Scholar, and screening the reference lists of related review articles. Forty-two papers were read in full text, and one paper was found to be relevant for inclusion. Therefore, a total of twelve studies were included for quality appraisal.

### Eligibility criteria

We used the following inclusion criteria: peer-reviewed scientific studies published in English, Norwegian, Danish, or Swedish between 2011 and 2022; qualitative studies and qualitative parts of mixed method studies with any design illuminating the perspective of the rural family caregivers for an adult person at any age with cancer at the end of life; studies exploring multiple perspectives if the family caregivers’ perspective could be separated from others’ perspectives; and studies in which the majority of caregivers were providing care for a person suffering from cancer. Relevant studies were included no matter what the relationship to the care recipient was as long as they were defined as family caregivers by the participants in the included studies. We excluded studies if the participants did not live in rural areas or did not care for the ill person at home.

### Qualitative appraisal of the studies

The first author critically appraised the research papers relevant for inclusion using the Joanna Briggs Institute Critical Appraisal tools (JBI) [37] in agreement with the third author. This ten-criteria tool evaluates the congruency between aim, methodology, data collection, analysis method and philosophical perspective [37]. It has been assessed as a reliable resource for evaluating qualitative research efficiently. However, it has been argued that some aspects of qualitative research are difficult to appraise and therefore depend on subjective judgement [39]. We particularly focused on rich findings and taking care of ethical aspects. No studies were excluded based on quality assessment (Table 1).

**Table 1:** Summary of the JBI critical appraisal of qualitative evidence for the included studies

Quality appraisal	Adejoh (2021) [40]	Barlund (2021) [41]	Duggleby (2011) [42]	Githaiga (2017) [43]	Gunn (2022) [44]	Hansen (2012) [45]	Johnston (2012) [46]	Marsh (2019) [47]	Rainsford (2018) [48]	Robinson (2012) [49]	Spelten (2019) [50]	Williams (2013) [51]
Congruity between the stated philosophical perspective and the research methodology	Yes	Yes	Yes	Yes	Yes	Yes	Yes	Yes	Yes	Yes	Yes	Yes
Congruity between the research methodology and the research question or objectives	Yes	Yes	Yes	Yes	Yes	Yes	Yes	Yes	Yes	Yes	Yes	Yes
Congruity between the research methodology and the methods used to collect data	Yes	Yes	Yes	Yes	Yes	Yes	Yes	Yes	Yes	Yes	Yes	Yes
Congruity between the research methodology and the representation and analysis of data	Yes	Yes	Yes	Yes	Yes	Yes	Yes	Yes	Yes	Yes	Yes	Yes
Congruence between the research methodology and the interpretation of results	Yes	Yes	Yes	Yes	Yes	Yes	Yes	Yes	Yes	Yes	Yes	Yes
Declaration of the researcher’s cultural or theoretical orientation.	Yes	Yes	No cultural	No cultural	No cultural	Yes	No cultural	Yes	No cultural	Yes	No cultural	No
The influence of the researcher on the research, and vice versa, is addressed	Yes	Yes	Yes	Yes	Yes	Yes	Yes	Yes	Yes	No	No	Yes
Representation of participants and their voices.	Yes	Yes	Yes	Yes	Yes	Yes	Yes	Yes	Yes	Yes	Yes	Yes
Ethical approval by an appropriate body	Yes	Yes	Yes	Yes	Yes	Yes	Yes	Yes	Yes	Yes	Yes	Yes
Relationship between the conclusions of the study and the analysis or interpretation of the data	Yes	Yes	Yes	Yes	Yes	Yes	Yes	Yes	Yes	Yes	Yes	Yes
Overall appraisal	Included	Included	Included	Included	Included	Included	Included	Included	Included	Included	Include	Include

### Data extraction, translation and synthesis

Characteristics and key contextual information from the included studies were extracted. Data about rural family caregivers’ experience of home-based end-of-life care for cancer care recipients deriving from each study were collected in a tabular form in an Excel spreadsheet and broken down into first-order constructs (quotes from participants) and second-order constructs (the primary author´s interpretations) [32]. According to Noblit & Hare [30], definitions used by the original authors, such as metaphors, concepts, phrases and ideas, should be a focus.

During the process of reciprocal synthesis (when concepts in one study can incorporate those of another), definitions from all twelve studies were compared to and translated into each other. Synthesizing translations is defined as the process of going beyond the findings of the individual study, “making a whole of something more than parts alone” [30]. Similarities between the findings of the included studies supported our reciprocal translation. Although no refutational concepts were found, we documented the differences in participants’ perspectives, demonstrating the diversity of experiences in the included studies.

Finally, categories of shared meaning that could answer the research question were clustered and synthesized into third-order themes. All three researchers collaborated in developing the third-order themes. A line of argument synthesis expressed as a metaphorical phrase was created from these themes, representing a novel interpretation of our findings. The same user representative was consulted during this process. This procedure, together with the authors’ rich experiences in the field of family caregiving and rural family caregiving, facilitated the novel interpretation utilized by the current study.

## Results

The twelve included studies on family caregivers’ experiences either applied grounded theory, ethnography, phenomenology or narrative enquiry or used a descriptive or explorative design. Except for William et al.’s [51] study, which explored daily journal entries, the data-collection process comprised individual interviews or focus groups. The qualitative parts of two mixed methods studies were included [49, 51]. The studies were published between 2011 and 2022 and represented the following nine countries: Nigeria, Uganda, and Zimbabwe (*n* = 1) and Kenya (*n* = 1), Australia (*n* = 4), Canada (*n* = 3), Norway (*n* = 1), Scotland (*n* = 1) and the USA (*n* = 1).

Altogether, 240 family caregivers were included in the studies. Four articles included caregivers of either persons with cancer or those with other life-limiting illnesses [45, 47–49]. Participants in Hansen et al.’s [45] study were family members of persons with different life-threatening illnesses, where 52 percent had cancer. In the studies of Robinson et al. [49] and Marsh et al. [47], all care recipients had life-limiting diseases, while in Rainford et al.’s [48] study, most of the care recipients had cancer. A consultation with the first author of Marsh et al.’s [47] study revealed that not all family caregivers had known the care recipients’ diagnoses, but at least seven care recipients had died of cancer. The findings in Robinson et al.’s [49] study revealed that many care recipients suffered from cancer. Gender was not described in all studies. A total of 179 family caregivers for cancer patients were identified, including 32 men, 108 women, and 39 individuals (gender is unknown). The age of the participants ranged between 18 and 94 years. Three studies did not describe the age of the caregivers [46, 48, 50]. In seven articles, the time of caregiving ranged from two months to twelve years caring for a person with cancer. In five studies, the time range was not described [40, 41, 46–48].

Four studies included family caregivers’ perspectives, as well as health care personnel’s and care recipient’s perspectives [42, 46, 48, 50]. In all these studies, clear distinctions were made between the family caregivers’ perspective and others’ perspectives, making it possible to include only caregivers’ experiences in our study. Six of the twelve studies included only bereaved caregivers [41–44, 47, 49], while two included both family caregivers and beavered caregivers [43, 44]. In four studies, close friends had been defined as family caregivers [40, 42, 46, 49], while another four studies [40, 42, 47, 50] did not specify family/next-of-kin relationships. Family caregivers of persons with cancer in the study from Kenya [43] were from central Kenya and rural homes, and we included only findings from the rural families. An overview of the studies is presented in Table 2.

**Table 2:** Overview of included studies

Study (author/ publication years/country of origin)	Aim	Research design	Method	Setting & sample	Data analysis	Major findings
Adejoh et al., 2021 [40] Africa South of Sahara	To understand the role, impact, and support of informal caregivers of patients with advanced cancer when interacting with palliative care services in Nigeria, Uganda, and Zimbabwe	Qualitative research	Semi-structured interviews	48 participants informal caregivers of patients with advanced cancer (equal numbers of men and women) Age 19–75 Caregiver: Friend, partner, ex-partner, sibling, parent, adult child, and other relatives	Qualitative secondary analysis using a framework approach to thematic analysis	Five themes: Caregivers are coordinators of emotional, practical, and health service matters; caregiving comes at a personal social and financial cost; practical and emotional support received and required; experience of interacting and liaising with palliative care services; and barriers and recommendations relating to the involvement of palliative care
Barlund et al., 2021 [41] Norway Rural region of Sogn og Fjordane	To explore factors that determined the feeling of security of caregivers of dying patients with advanced cancer who cared for the patient at home at the end of life in the region of Sogn og Fjordane in Norway	Qualitative retrospective study	Semi-structured in-depth interviews	10 female bereaved caregivers from nine families with experience caring for cancer patients at home at the end of life Age 41–76 Caregiver: Parent, adult child, and spouse	Thematic analysis following Kvale and Brinkmann’s analysis	Three themes: Personal factors; healthcare professionals; and organization of healthcare
Duggleby et al., 2011 [42] Canada Three rural health regions in a Western Canadian province	To explore the context in which older rural patients receiving palliative care and their families experience transitions	Grounded theory study	Qualitative Open-ended telephone interviews with older cancer patients and bereaved family caregivers Four focus groups with healthcare- personnel	10 family members within the first year of bereavement after providing care to an older family member (> 60) with advanced cancer (8 female, 2 males) Age 18 or > 18 Caregiver: Defined broadly as family or friends	Thorne’s interpretive description qualitative analysis approach	Four themes: Community connectedness/ isolation; lack of accessibility to care; communication and information issues; and independence/ dependence
Githaiga, 2017 [43] Kenya Nairobi	Explores the experiences of a small group of Nairobi women caring for a family cancer patient at home	Qualitative research	Semi-structured in-depth interviews and four mini-focus groups	20 female family caregivers and bereaved family caregivers for cancer patients at home Age 27–75 Caregiver: Sister, mother, and daughter	Interpretative phenomenological analysis	Two themes: Role reversal in parental caregiving; and patriarchal caregiving protocols in marital homes
Gunn et al., 2022 [44] Australia Rural part	To explore the experiences of people caring for someone with cancer, while living in rural Australia, and the impact of the cancer-caring role on their wellbeing	Qualitative phenomenological approach, underpinned by an essentialist epistemology	Semi-structured telephone- interviews	18 adults in regional or remote Australia who cared for a person with cancer at home (12 female, 6 male) Aged 32–77 Caregiver: Spouse, adult daughter, and mother	Braun and Clarke’s thematic analysis	Eight themes: Travel is hard but supports are available; frustration with systems that do not demonstrate understanding of the rural context; the importance of lay and peer support; the impact of access to trusted, local health care services; the importance of access to rurally relevant information; living with uncertainty and balancing loss with hope; reluctance to seek or accept psychological support; and the gendered nature of care
Hansen et al., 2012 [45] USA Rural agricultural Pacific Northwest County	To describe the perspectives of primary family caregivers regarding experiences with formal and informal care at the end of life for dying older adults in one rural agricultural county	Qualitative descriptive study	Open-ended, semi structured interviews	23 primary caregivers for a decedent older than 60 who died at home (16 female, 7 male) Caregiver: Spouse or partner, adult child and relatives	Qualitative description guided analysis of the data	Two themes: Benefits to the End-of-Life Care Experience (formal care and Informal Care); and Challenges to the End-of-Life Care Experience (formal care and Informal Care)
Johnston et al., 2012 [46] Scotland Highlands and West of Scotland including rural, remote, and socially deprived areas	To understand patient and caregiver experiences of end-of-life care and to explore how patients care for themselves at the end of life in Scotland	Qualitative study	In-depth unstructured serial triangulated interviews	19 main caregivers for cancer patients at the end of life Caregiver: Wife, husband, daughter, father and friends	Framework analysis—thematic qualitative analysis	Six themes: Maintaining normality; preparing for death; support from family and friends; self-care strategies physical; Selfcare strategies emotional; and support from healthcare professionals
Marsh et al., 2019 Australia [47] A small community in rural Tasmania community	To explore experiences of end‐of‐life care in rural community	Descriptive qualitative study	Semi‐structured, in‐depth interviews	18 bereaved former caregivers cared for someone who had died from a life‐limiting illness within the previous 3 years or who were still caring for someone in the advanced stages of a life‐limiting illness Age: All but four were over retirement age	Thematic analysis	Six themes: The various supports that people utilized to die at home; issues of isolation; the impacts that difficult relationships can have on the dying experience; experiences of losing or maintaining control; talking about death and dying; and the variations of grief and bereavement
Rainsford et al., 2018 [48] Australia Snowy Monaro region of New South Wales	To explore the concept of the “good death” articulated by rural patients with life-limiting illnesses, and their family caregivers	Ethnographic study	Open-ended interviews, observations, and field notes form	18 family caregivers to rural patients with life-limiting illnesses (10 female, 8 male) Caregiver: Wife, husband or partner, daughter, son, brother, and sister	Semantic analysis of narratives	Three themes: a connection with one’s previous identity; autonomy and control over decisions regarding management of end-of-life care; and not being overwhelmed by the physical management of the dying process
Robinson et al., 2012 [49] Canada Rural part	To understand the ways in which we can support the wellbeing of family caregivers of rural palliative patients, with focus on their own needs and self-care	Mixed method Study with questionnaires and interviews	Semi-structured telephone interviews gathering in-depth narratives	23 family caregivers who had cared for a family member who had died at home in a rural area (20 female, 3 male) Age 36–65 and > 65 (12 persons) Caregiver: Partner, sibling, adult child, or friend	Constant comparative analyses in the qualitative part of the study	Four themes: The need to be(come) a palliative caregiver; the need to be skilled and know more; the need to navigate competing wishes, needs, demands, and priorities; and the need for “an extra pair of hands”
Spelten et al., 2019 Australia [50] A rural town in Northern Victoria	To describe the experience of families and nurses with extended rural palliative care to support dying at home	Qualitative research with a phenomenological approach	Semi-structured interviews	10 family members of 17 cancer patients with extended rural palliative care for a family member dying at home (9 female, 1 male) Caregivers: Spouse or adult child	Descriptive analysis	Two themes: The palliative care services (service and nurses); and the process of dying at home (unfamiliarity, positive experiences, support after death, and positive impact on bereavement)
Williams et al., 2013 [51] Canada Rural Western Canada	To explore the hopes and challenges of rural female family caregivers of persons with advanced cancer	Mixed method study. Narrative enquiry approach in the qualitative part	Daily journal entries documenting narratives	23 rural female family caregivers of persons with advanced cancer Age18 or > 18 Caregiver: Spouse, daughter, and mother	Cortazzi’s method for narrative analysis	Four themes: Hope; practical and emotional challenges; self-care strategies; and the emotional journey

Based on the translation and synthesis of the findings in the 12 studies, we developed the following four themes: providing family care at the end of life in rural areas—a challenging endeavour; the burdening responsibility of rural caregiving—a lonesome experience; working on and behind the scenes; and the strong and weak spots of community connectedness in rural areas.

### Providing family care at the end of life in rural areas—a challenging endeavour

Family care in rural areas was found to be affected by the lack of availability and continuity of community healthcare services and the long distances to specialized healthcare facilities. Access to palliative care for rural families was strongly linked to barriers related to long distances [40–42, 44, 45, 47, 49], the poor quality of roads [40, 41, 45, 47, 49], bad weather with closed roads [40, 41, 49], a lack of public transport [40, 41, 44, 47], not owning a car [40, 41], defective working conditions of one’s car [44] and caregivers’ lack of a driver’s licence [41] and fuel shortages in the country and toll- free phones [40]. Economic reasons were mentioned as a main hindrance to accessing palliative care and essential medication [40].

Remote and scarcely populated areas had poor access to medical services and equipment, especially at night and on weekends [40, 45, 47, 49, 50]. In addition, out-of-hours prescriptions became a problem due to the shortage of local doctors [48]. Due to long distances, health professionals were not able to respond quickly enough [41, 44, 45, 50]. These factors contributed to caregivers’ feelings of insecurity and loss of control [50]. When the family’s youngest members moved away from their rural homes and were no longer available daily, caregivers felt the absence of support [45, 47, 51]. However, members of the extended family living in other parts of the country sometimes moved to the caregivers’ area to help [44, 47]. Nonetheless, there was evidence that some of the caregivers did not receive support from their own families [51]: “*I guess my nerves are frayed – I don’t seem to get too much support from my family – and I don’t have anyone to talk to and cry on their shoulder – my support person is dying – where is my hope?”* [51].

Sometimes using public transport was a bad choice due to the poor health condition of the ill family member: [40, 47]: “…*For her to get out of the house and board an omnibus it’s hard, she would be in pain like when the omnibus is moving and she gets shaken, the pain increases…”* [40]. Several caregivers found it difficult to afford additional transportation and accommodation costs [40, 42, 44, 47, 49].

Scheduling palliative treatment in the nearest city was reported to be time-consuming and stressful for family caregivers who had to arrange long-distance travel and leave behind other family obligations [41–47, 49, 51]. In some cases, family caregivers attended appointments on the ill family member’s behalf because their advanced illness made travelling impossible [40]. Some felt frustrated when healthcare personnel did not understand their rural context and the difficulties related to escorting their ill family member on long journeys [41, 44]. Some families even declined specialist treatment [45, 47] due to exhausting travel being required. Others were lacking information about available services [40].

Lack of services or inadequate and poorly organized services was found to put pressure on families providing end-of-life care [40–42, 45, 47–50]. The caregivers experienced healthcare services to be fragmented [42, 47, 49]; furthermore, when seeking specialized palliative care support, they were passed back and forth between services. Sometimes they did not get help at all [47], and some patients had to be transferred to local care facilities [41, 48]. The shortage of available and especially trained healthcare providers left caregivers feeling unsupported and distressed [41, 42, 45, 47, 49]. They complained about the shortage of health care providers’ competence in relieving pain and other symptoms [42, 45, 47]: *“So, there was no palliative care…Who provides the help? That’s all we wanted to know and then everybody kept saying, “well we only do this bit though…”* [47]. Language barriers often compounded the situation [40, 41, 50],*” …if he said something, they answered “yes” no matter if they understood or not. So, both he* [i.e., the patient] *and I felt terribly insecure …”* [41].

Long distances and a lack of resources were found to hinder the continuity of treatment and care [40, 42, 44, 45, 47, 49, 51]. Caregivers reported having to constantly repeat important information to unfamiliar health care providers [40, 42]: “… *had three physicians come and go and then there was a fourth one…”* [42]. Family caregivers regretted getting lost on their way through the healthcare system and wished for information about how to access palliative care and financial support [40, 44, 45, 49, 50].

### The burdening responsibility of rural caregiving – a lonesome experience

Both healthcare personnel and the care recipients expected rural family caregivers to take on the caregiver role [41, 49]. For the caregivers, it was seen as unacceptable to transfer their ill family member to institutional care [44, 48, 49]. Adult children, particularly women, often felt a moral obligation to care for their ill family members at the end of life [40, 43–45, 49]. However, in some African countries, this responsibility was challenging due to traditional patriarchal gender roles and specific cultural practices [43]: *“And there’re things which I think children should not do for mothers; it is seeing their nakedness, but we had to…can you imagine holding your mother’s leg wide open so that the nurse can put in the catheter? … You can see she’s resisting from body language [silence], but there you are…”* [43].

Although family caregivers emphasized that they had taken on their role voluntarily, dwelling in rural areas and handling demanding issues was perceived as a lonesome experience and an enormous responsibility they had not been prepared for [40–42, 47, 49, 51]: *“Well, all the responsibility was on me. That’s the way I felt … they were basically just coming here to take his blood pressure and to see how he was feeling today. But everything else was, was in my hands to look after him. I just felt so alone in this situation that I had… I really struggled with it”* [42]. Relationships could turn out to be demanding if the ill person’s anger was directed at the family caregiver [41, 42]. This added to the burden of loneliness felt by rural caregivers [41, 43, 47, 49, 51]. The family caregivers were concerned that healthcare providers would doubt their ability as caregivers; thus, they sometimes agreed to do tasks that they truly did not want to do, such as administering medication and medical procedures: “… *I don’t want to do this.” And every time they’d* [RNs] *come, they kept saying I had to. And it almost was as if it was a power of wills. And finally... in the morning I got up, I got his needle ready, and I gave him his needle and I figured, okay you won. But that still bothers me* [voice shaking]” [49].

Being the main caregiver was often reported to mean “*learning as you fly*” ([49], p. 477). Feeling unprepared for their new role [40, 41, 44–51], caregivers reported struggling with conflicting emotions of loss, grief, and hope for the future [44, 51]: “*But I can choose to hope. There may be light at the back of the tunnel yet – every once in a while, it sneaks in when I’m not looking”* [51]. The lack of emotional and practical support in the end stages of life was perceived as exhausting [41, 43–46, 49, 50]. Caregivers expressed the need to discuss the ill family member’s condition with health personnel, and they desired validation of their care competence [40–42, 46–51]. In addition, they longed for support and the possibility of sharing their concerns with someone who was familiar with their situation [41, 42, 46, 47]: “*… I get very depressed sometimes… The nurses come but… they don’t ask how I am they’re more interested in* [Patient]*… There’s nobody who’s really told us what to expect…”* [46]. Family caregivers also reported missing discussing death and dying more openly with health care providers [40, 41, 44, 45, 47, 49]. They expressed that openness, and a shared understanding of the situation would make them feel more confident in managing end-of-life care [40, 41, 44].

As family caregivers reported lacking knowledge about end-of-life care and dying, it was difficult for them to observe significant signs and to make care-related decisions [40, 42, 44, 45, 47, 50]. Gaps of knowledge and the inability to alleviate suffering [41, 42, 45, 46, 49] contributed to a lack of self-confidence in caregivers: *“All these changes, right?* …*It’s a process. When you haven’t been through it before, you don’t know what it is…”* [41]. Sometimes, not knowing enough hindered caregivers from making informed decisions [40–42, 49] or reaching out to healthcare professionals in time [40, 46, 47, 50]: *“Dr. A always told me to, um, get palliative care. But I didn’t want it, cause to me, palliative care is when they are dying. And X was not dying…”* [50]. Others reported feeling responsible for sharing their observations with healthcare personnel to prevent missing signs of deterioration [41, 45], and they were disappointed if health care providers did not respond in time: *“It felt safe and secure for us to know that they were visiting her* [i.e. home nursing care]. *But when changes occurred, they were slow. And in that phase of changing, it’s very tough to be a caregiver, because you see things, feel things and know things, and the rest* [i.e. HCP] *doesn’t follow”* [41]. Although family caregivers reported experiencing caregiving as challenging and burdensome, they did not exhibit an inability or reluctance to provide care [44, 48, 49]. They aimed to maintain their independence and were reluctant to ask for help, which made them feel more alone [42].

### Working on and behind the scenes

The role of being a family caregiver required extensive work to enable one’s family members to remain at home for as long as possible [40, 41, 45–49]. Some careers referred to this work as “shadow work” or “behind the scenes work” ([49], p.477). “Shadow work” was reported as being present in various aspects of daily life, including helping ill family members engage in familiar activities, carrying out spiritual and religious tasks, maintaining social connections and enjoying nature [46, 47, 50]. Additionally, caregivers also reported taking care of rural properties and farms and performing household chores and other activities that their ill family members were no longer able to do [40, 46, 47, 49].

Some caregivers felt as though they were becoming a “mininurse” [49], providing and coordinating care around the clock [40, 41, 44, 45, 49]. They had to provide emotional support to prevent anxiety and agitation, assist with personal hygiene, manage nasogastric tubes, provide physical therapy, and follow extensive pain and symptom control regimens [40, 49]. For family caregivers with a background in healthcare, the role could be especially challenging due to high expectations from family members and healthcare providers [45, 49]: *“… But the demands of having to answer all the medical questions cause I’m the nurse in the family, having to take care of all the medical issues, and then to physically take her to appointments and stuff… How did I do it? I don’t know, you just do it I guess, I don’t even know how you figure out to do it*…” [45].

These core tasks and activities often made caregivers neglect their own needs, particularly seeking help [44, 48, 49]. As a result, caregivers reported forgetting about their own physical, social and mental health and well-being [41, 42, 44, 46, 47] as they prioritized the ill family member’s needs before their own: “*Oh, I just didn’t pay attention to caring for myself… I was just focused on my mom. But I have a good husband and a good marriage, and so that was good. I was focused on the others in my family* [49]. In addition, caregivers reported that it was difficult to maintain their network and former activities when all their available time had to be spent on caregiving and engaging in shadow work [47, 49, 51]. Some caregivers were even forced to give up their jobs to provide care, which had far-reaching consequences for the whole family’s financial situation [40]: *“…I have to withdraw the children from private school…we were not able to pay accommodation, we have to lodge for two years. The landlord took over, took possession of the house; then we had to go to friends, my load as I speak is still spread outside…*” [40].

Care for young children, household chores, and work life was difficult to balance with the palliative care required for ill family members [40, 43, 47–49, 51]. In addition, some family caregivers had to deal with their own health problems [40, 41, 45, 51]. Marital conflicts reportedly arose when female caregivers prioritized caring for a terminally ill relative and spending less time on other family obligations [43]. When a break was available, such time was usually used for basic self-care, “*… When friends would come over so that I had a minute to go and get dressed or have a shower, that was what allowed me to look after myself enough to keep going”* [49]. Only a few family caregivers took the time to seek professional counselling to uphold their spirit [51].

### The strong and weak spots of community connectedness in rural areas

For the most, caregivers wanted to fulfil their family member’s wish to die in their rural environment [40, 41, 44–49, 51]. Rural palliative care reportedly provided a sense of closeness, social connectedness, and higher quality when supported by professionals who were familiar with and appreciated by the families [41, 42, 45, 47, 49]. Caregivers viewed the home as a safe and peaceful place for end-of-life care and death [40–43, 47, 48, 50, 51] if formal and informal care was available and friends and family members were present [48]. When caring for their loved ones at home, caregivers reported feeling in control, and there was no need to worry about hospital protocols and visiting hours: [Home is] *a controlled space that* [we] *have control of, where as soon as we go into a hospital, we’re in somebody else’s controlled space* [48]. Feeling supported brought hope to the lives of caregivers [44, 51] and combated their feeling of loneliness: [51].

When palliative care teams were involved early in the illness trajectory, family caregivers felt particularly secure [41]. However, as the illness progressed, some caregivers became overwhelmed by responsibility, and the familiar, safe space of home could become an unsafe space [40, 41, 48]. Due to demanding caring tasks, some caregivers had to transfer their ill family member to a nursing home or to a rural hospital [43, 48]: This triggered feelings of shame or guilt in family caregivers, as their own interests took precedence over those of the ill person [43, 48, 51]. However, caregivers expressed that moving to a local facility did not negatively impact the quality of end-of-life care of their loved one because they knew the staff at the local hospitals [41, 48].

Having access to trustworthy and well-organized local treatment options, such as specialized cancer teams, cancer nurse coordinators and teleconsultations made family caregivers feel more confident in providing palliative care at home [40, 41, 44, 45, 47, 48, 50]. Nurses who organized homecare in a responsive and efficient manner [41, 44, 45, 47, 50], sometimes out-of-hours, provided a sense of security and made caregivers feel in control of the situation [47]. Due to healthcare providers’ familiarity with the family, they were willing to disregard rules and policies to provide person-centred end-of-life care compassionately [41, 42, 45, 50]. “[The local staff] … *were brilliant … and … the compassion from the staff was amazing.* [The GP] *put his arm around* [my partner] *one day and said, «I love you,* [X]” *and that just meant so much to him, you know?*” [47]. Interaction with labour and social services was also reported as being important for caregivers’ sense of security [41].

Despite the connectedness of caregivers and health professionals described in rural communities, a lack of privacy and a crossing of personal boundaries could occur [45, 47, 49]. While some families perceived the professional caregivers as friends [45, 49], others worried about their privacy and did not want any contact with health care providers they knew [49]. The shortness of staff in rural areas gave caregivers little choice if they disliked the healthcare providers or questioned their ability to provide care [41, 42, 44, 45].

Family, friends, and neighbours were perceived as playing an essential role in closing rural healthcare gaps [49]. Their support was described as a distinctive feature of rural life and was perceived as especially valuable [40, 42–47, 49, 51] when it allowed caregivers to take a break [44, 49]: …*if I was finding it hard with [first name] I could ring one of the others and have a talk to get it of your shoulders… It’s a relief group more than anything*” [44].

Such informal networks reportedly provided families with counselling to reduce the psychological impact of cancer and, for some, also provided financial support through fundraising for medical treatment and care needs [40, 45]. Laypeople with a medical background helped with decision-making and assisted in finding resources or obtaining information about, e.g., the use of formal systems [45]. Neighbours helped with household chores and provided a range of “shadow work” [48, 49], e.g., managing farm property [45, 48, 49] and supplying wood: Knowing that neighbours would respond to any call for help was associated with caregivers’ perceptions of “dying safely” [48, 50]. “*…and during the night… I could contact them for reassurance. …we had people coming and playing music and just… we were singing, … and comforting him… absolutely the environment he wanted”* [50].

For some family caregivers support groups [44, 47] and religious and spiritual activities that could foster their hope were available in the communities [40, 43, 45, 46–51]. In some African countries, caregivers depended on community fundraising to ensure medical treatment [40]. Family caregivers found comfort in the continued support of their community even after their loved one’s death [45, 47–49].

### A line of argument synthesis: ambivalent and heavy burdened wanderers on a road less travelled

In our line of argument synthesis, we expressed the meaning of family end-of-life cancer care in rural and remote areas by the metaphorical phrase “ambivalent and heavy burdened wanderers on a road less travelled”. A road less travelled represents the symbolic meaning of facing a new and unfamiliar role by trying to safeguard an ill loved one at the end stage of life. The metaphor also represents the poorly conditioned roads in rural and remote areas, far away from specialized palliative care services. The caregivers were unfamiliar with their new role, and they lacked knowledge, competence, and professional support. Due to these holes and cracks in the road that impeded their passage, it was difficult for them to move on safely.

Nevertheless, caregivers took on the risk of wandering these unfamiliar roads. They took on an enormous burden of responsibility when trying to protect their loved ones in the sparseness or absence of professional support. Their load was doubled by working on the scene by engaging in caring tasks and working behind the scenes in ways that were invisible to others, for instance, by organizing and following their loved ones on long-distance journeys to specialized health care services. In addition, taking over roles from their ill family member and combining other family and work obligations with caregiving tasks added to their burden and made them neglect their own needs.

Their wandering on a road less travelled was hallmarked by ambivalence and conflicting feelings. On the one hand, caregivers perceived home as a safe and controlled space and went all in to fulfil their loved ones’ wish to die at home. On the other hand, home could turn into an unsafe space filled with loneliness and hopelessness when caregivers were left feeling unsupported by health professionals or family members. Although the compliant support of nearby community healthcare services was highly appreciated, caregivers had little choice if the few available healthcare workers could not fulfil their expectations of professional or relational competence. Being familiar with the local professionals could feel supportive, but caregivers were ambivalent about sharing private matters with healthcare workers who were well known within their small communities.

Neighbours, friends, and dedicated family members represented a safety barrier on the road on which the heavy burdened wanderers travelled. Knowing that they could turn to community support if needed made it easier for them to continue down the road more safely while striving to fulfil their loved ones’ wishes to die at home.

## Discussion

The purpose of this study was to synthesize and reinterpret findings from qualitative studies that focus on the experiences of rural family caregivers of adult persons with cancer at the end of life. In our line of argument synthesis, we present a novel interpretation that emphasizes the meaning of caring responsibilities in rural areas, which is expressed by the metaphorical phrase “ambivalent and heavy burdened wanderers on a road less travelled”. Family care in rural areas was found to be affected by the lack of availability and continuity of community healthcare services and the long distances to specialized health care services. Family caregivers reported feeling alone and overwhelmed by the enormous responsibility of taking care of their loved ones [40, 41, 43, 47, 49, 51]. The caregivers examined in our meta-ethnography had to manage tasks such as administering medication and using specialized equipment when providing end-of-life care. Although such unwanted tasks were sometimes imposed upon them, they decided to overcome those obstacles and carry on. However, not being trained to perform complicated tasks may jeopardize the care recipients’ well-being or even their safety. Devik et al. [52] reported that family caregivers take over tasks because they mistrust professional caregivers’ ability to follow up on palliative care. Fjose et al. [53] found that family caregivers without medical training are unable to follow up, and they do not know whom to contact. However, the caregivers in our study acknowledged their limitations and need for professional support [43, 48, 49, 51]. Unfortunately, their needs could not always be fulfilled due to the shortness of available professionals and their lack of competence in palliative end-of-life care. Feeling unprepared for their new role as caregivers and lacking information and knowledge were some of the greatest obstacles reported by those walking on this less-travelled road. The feeling of being unprepared for the caregiver role is in line with other studies on end-of-life care and is not limited to rural areas [7, 20, 54, 55]. Not knowing what to expect around the next bend of the road while carrying the heavy load of responsibility is interpreted as a double burden in our meta-ethnography.

Communication between community healthcare personnel and family caregivers was found to be challenging due to the high turnover of professionals, as well as to language barriers. Caregivers reported feeling anxious and insecure due to such barriers [40–42, 50]. This issue has also been highlighted in a previous study, which found that families face unpredictability due to constantly changing professionals who are unfamiliar with the family’s needs [53]. According to our research, caregivers often felt they were being passed around between various services in their effort to receive community-based palliative care for their loved ones [47]. This phenomenon, known as being “handballed”([56], p.18), is often attributed to poor communication between healthcare services [56, 57] and a lack of information sharing between hospitals and community settings [57]. Our research shows that the shortage of primary health care personnel in communities trained in palliative care added to caregivers’ reported feelings of unsafety [45, 47, 49]. This scarcity of resources and lack of interprofessional teamwork pose significant obstacles to providing palliative care in rural areas [14, 58–60], which in turns raises concerns about patient safety [18, 61]. The responsibility to provide high-quality care made caregivers feel alone [45, 47, 49]. Gott et al. [62] found that elderly individuals’ family caregivers receive limited community support in end-of-life care. They often felt isolated and lonely in their caregiving role, with no one to spend time with, which causes them to feel lonely. Limited access to palliative care services in rural areas results in family members having to provide such care, which can negatively impact their own well-being [15]. Given the demographic shift towards community-based palliative care, the existing shortage of healthcare workers in rural areas is likely to worsen [4].

Our meta-ethnography shows that some family caregivers who have provided palliative care for their dying loved ones considered doing so a rewarding and voluntary task [41, 48, 51]. However, others felt obliged to provide end-of-life care to their family members due to emotional, social, and cultural reasons [43–45, 49]. This finding is in agreement with results of Zuh et al.'s [10] study, which showed that family members often feel morally bound to provide palliative care, with little or no choice in the matter. Our findings indicate that rural caregivers in developing countries encounter extra difficulties related to cultural norms and gender roles. Providing intimate care tasks for parents or relatives of the same gender often triggered feelings of guilt and shame. In cases where married women cared for relatives in their in-laws’ homes, approval from the male head of the household was required. Similarly, Gambe et al. found in a systematic review that cultural and gender norms add a significant burden for female caregivers in Sub-Saharan Africa [28].

The participants in our study faced significant challenges in fulfilling their role as family caregivers, which came at personal and social costs [40–42, 44, 46, 49, 51]. Their caregiving responsibilities were in place 24/7 [40, 41, 44, 49], and dealing with them left the caregivers feeling exhausted and lonely when they felt unsupported [40, 41, 44, 45, 49, 50]. Caregivers struggled to balance emotional support and caregiving tasks with household chores and work obligations [40, 49], which often led to them neglecting their own needs and health. This is consistent with Holland’s [55] study, which suggested that caregiving can disrupt the balance between work, personal space and time and be seen as a job. Other research has shown that providing palliative care can result in caregiver strain and poor physical and mental health [9, 15, 17, 19, 63, 64]. The findings from a cross-sectional survey study indicated that family caregivers’ psychological health may be even more affected than that of care recipients [23]. Furthermore, family caregivers living in rural areas are a vulnerable population because there is a lack of health care services to turn to when struggling with their own health [9]. Finding a balance between the care recipients’ needs and self-care is difficult, and caregivers often face moral dilemmas when prioritizing their own needs relative to their loved ones’ well-being.

According to the findings of our meta-ethnography, family caregivers in rural areas provide extensive work behind the scenes, which is referred to as “shadow work” ([49], p.477) or invisible work [48], in addition to their caregiving responsibilities. Accompanying an ill loved one to appointments at far away specialized healthcare services reportedly adds to the burden of family caregivers. Rural caregivers expressed disappointment because urban healthcare professionals lacked an understanding of the rural context and its challenges. Additionally, studies have shown that end-of-life caregiving involves invisible forms of labour that go beyond caring tasks [55, 65]. Wiles et al. [65] argued that rather than solely focusing on the burdens faced by caregivers in rural areas, it is important to also understand the invisible labour involved in coordinating and managing care at home. Holland [55] suggested that healthcare workers should recognize the invisible work of family caregivers, which is an essential part of care and a valuable contribution to the formal health system. In our study, we found that juggling caregiving tasks along with family obligations, work, and the logistics of rural living were interpreted as another double burden for caregivers.

Long-distance travel to centralized healthcare services was reported to be challenging, and families sometimes declined palliative treatment due to exhaustion [47, 52]. This finding is in accordance with Zullig’s results, which indicated that exhausting travels could result in missed appointments and inadequate follow-up care [66]. Additionally, other studies have revealed that the financial burden associated with travelling long distances to receive care is a significant obstacle [67]. A household with a low annual income can have transportation-related barriers that limit access to appropriate palliative care [4]. Family caregivers and their ill family members sometimes hesitate to relocate for treatment, even if it means missing out on potentially beneficial or lifesaving care because such a relocation can place a strain on their support networks [16]. Studies have also found that patients who must travel long distances to receive palliative care may end up dying in the hospital rather than at home, which is often their preference [4, 64].

Our meta-synthesis revealed that caregiving led to significant financial consequences for some families in Africa, including loss of income, disruption to children’s education, and even homelessness. Financial burden emerged as a significant stress factor, and a finding echoed in most of the 31 African studies reviewed by Gambe et al. [28].

The study revealed that community connectedness was viewed as both a source of support and a safety barrier for caregivers; however, it could also lead to ambivalence. Local healthcare workers were sometimes seen as friends; however, at other times, caregivers felt hesitant to share personal matters with people they knew in the community [41, 44, 45, 49]. Additionally, family caregivers were hesitant to raise concerns or voice their dissatisfaction if they perceived negative attitudes from healthcare workers or if they found the quality of care to be unsatisfactory [49]. Research has shown that caring for a dying family member can be difficult, especially with regard to issues of intimacy and privacy. This underscores the importance of creating a climate that allows family caregivers to express their thoughts and feelings [67]. According to Fjose et al. [53], family caregivers often experience tense relationships with healthcare professionals when care planning does not align with the care recipient’s wishes, thereby adding to the caregiver’s sense of insecurity.

The findings in our study show that the support of laypeople as neighbours and friends providing practical support was essential to caregivers’ relief and eased their feelings of loneliness. Robinson et al. [49] stated that informal community networks play an essential role in closing the healthcare gap. Not all caregivers in our study received the necessary support from their network during end-of-life care [45, 47, 51]. Woodman et al. [11] found that providing care at home is challenging if family members or close friends are uncomfortable with the realities of dying.

When caregivers have the support they need, they feel that they can provide a safe, peaceful and controllable environment for their loved ones who wish to die at home. Studies have shown that a personalized and comfortable environment at home is crucial for a peaceful end-of-life experience [13, 68]. Gerber et al. [12] and Auren-Møkleby et al. [69] highlighted that choosing to die at home is often a reflection of a person’s desire to maintain their sense of self, independence and autonomy. They also emphasized that end-of-life decisions are influenced by several personal, contextual and relational factors [12, 69].

### Strengths and limitations of the study

Our decision to conduct a meta-synthesis rather than another interview study was based on the amount of qualitative research detected and the lack of any synthesis of these studies. The strength of a meta-synthesis is to synthesize and reinterpret the included studies to inform policy makers, practice and research [32]. Although we used systematic search methods in four databases, guided by a specialized librarian, and applied several additional search methods, we may have missed studies. We followed the eMERGe guidance for reporting meta-ethnography [32] to increase the transparency, relevance, and quality of our research. The current study was registered in PROSPERO [33] to avoid the duplication of work. We used recommended tools such as the Raayan screening tool [38], Joanna Briggs quality assessments tool [37] and the Prisma flow diagram [35]. These tools have been used widely in systematic reviews and meta-synthesis research. Our search was limited to peer-reviewed journals written in English and Nordic languages published between January 2011 and December 2022 and we updated the searches continually prior to submission. Due to these inclusion criteria, there might be relevant studies that were not included in our meta-ethnography. Our decision not to include older studies was due to possible changes in the countries’ infrastructure and the availability of health care services. It has also been stated that the quality of a meta-ethnography is not dependent on the inclusion of all available studies, but to include enough studies to reach an in-depth interpretation [30]. We consider the involvement of a user representative as a strength. The contributions of this representative opened up a first-person perspective on the interpretation of the findings. The first and third authors were involved in the inclusion process. Articles identified for full text were read by both authors to determine if the studies met the inclusion criteria. The second author validated this process. All three researchers took part in the analysis procedures, the development of the themes and the line of argument synthesis. We consider it a strength that all authors have long clinical experience with family care for cancer patients. The second author is an academic cancer/oncology nurse and the head of a Regional Advisory Unit for Palliative Care. In addition, the third author has long experience conducting meta-synthesis research.

Including studies from four continents with different cultural, socioeconomic, and healthcare conditions might raise concerns about transferability. However, our findings reveal several common aspects of rural palliative caregiving across the studies we examined and the specific needs of caregivers in certain African countries. Illuminating the financial, cultural, and gender differences in rural family caregiving within developing countries may add valuable insights.

## Conclusions

Family caregivers in rural areas are facing numerous challenges, such as long distances to specialized health care services, harsh climates, poor road conditions and often poor quality of community-based end-of-life care. Caregiving combined with work-life and family obligations doubles family caregivers’ burden and makes them neglect their own needs. Our findings have implications for future health policies, clinical practice, and research. The financial consequences of caregiving in developing countries are alarming and should be addressed globally. Telehealth services, palliative beds in local nursing facilities, and palliative home care teams may relieve rural caregivers’ burden. Additionally, the family caregivers need emotional support, education and guidance from dedicated healthcare workers. Community connectedness and the support of laypeople may strengthen rural family caregivers; however, family caregivers’ need to talk openly with professionals outside their community should still be considered. Healthcare professionals in rural areas need to be well prepared to deliver high-quality end-of-life care. Digital lectures may bridge the existing gaps. Future research should design, and test interventions aimed at supporting rural caregivers’ well-being. Additionally, increased attention from healthcare authorities can make family caregivers’ tremendous contribution to palliative care more visible and appreciated.

## Data Availability

No datasets were generated or analysed during the current study.

## Abbreviations

EAPC: European Association for Palliative Care
WHO: World Health Organization
eMERGe: Evidence, Methodology, Ease, Resource, Geography and Economy-The meta-ethnography reporting guidance
PROSPERO: International Prospective Register of Systematic Reviews
PRISMA: Preferred Reporting Items for Systematic reviews and Meta-analyses
JBI: Joanna Briggs Institute Model of Evidence-Based Healthcare
PICo: Population, phenomenon of Interest, and Context (PICo) tool
